# Acute right extremity deep vein thrombosis and left-sided inferior vena cava thrombosis treated by percutaneous mechanical thrombectomy (PMT) combined with catheter directed thrombolysis (CDT): A case report

**DOI:** 10.1097/MD.0000000000037849

**Published:** 2024-04-19

**Authors:** Zhinan Ju, Wei Chen, Xixi Min, Kanghui Dai, Henan Zheng, Jiehua Qiu

**Affiliations:** a Department of Vascular Surgery, the Second Affiliated Hospital of Nanchang University, Nanchang, Jiangxi Province, P.R.China; b Medical College of Nanchang University, Nanchang, Jiangxi Province, P.R.China.

**Keywords:** acute deep vein thrombosis (DVT), anomaly, catheter directed thrombolysis (CDT), left-sided inferior vena cava (IVC), percutaneous mechanic thrombectomy (PMT)

## Abstract

**Introduction:**

Left-sided inferior vena cava (IVC) is an uncommon condition with a prevalence rate of 0.2% to 0.5%. Most of them remain asymptomatic and are discovered incidentally. The patient condition in this case is critical, and conventional procedures are not applicable. The surgical approach being considered is innovative, but it carries significant risks and uncertain therapeutic efficacy.

**Patient concerns:**

A 42-year-old male presented with acute right lower extremity pain with swelling for 2 days.

**Diagnosis:**

The patient was subsequently diagnosed with acute right lower extremity deep vein thrombosis, inferior vena cava thrombosis, and a left-sided IVC.

**Interventions:**

Based on the treatment guidelines for lower extremity deep venous thrombosis.

**Outcomes:**

We successfully cured him with percutaneous mechanic thrombectomy (PMT) combined with catheter directed thrombolysis (CDT).

**Conclusion and significance:**

The relatively low incidence of left-sided IVC does not diminish the significance of its identification. PMT combined with CDT is a safe way to treat acute thrombosis. It provides a new approach for similar patients in the future.

## 1. Introduction

Left-sided inferior vena cava (IVC) is an uncommon condition with a prevalence rate of 0.2% to 0.5%. Most of them remain asymptomatic and are discovered incidentally. Nevertheless, thrombotic complications in patients with left-sided IVC have been reported in a few cases. Herein, we describe a 42-year-old male who presented with acute right lower extremity pain with swelling for 2 days. The patient was subsequently diagnosed with acute right lower extremity deep vein thrombosis, inferior vena cava thrombosis, and a left-sided IVC.

## 2. Presentation of case

A 42-year-old man was admitted to our hospital through the emergency service with the chief complaint of severe pain in the right lower extremity for 2 days. He suffered a liver laceration in a car accident 2 weeks ago. Then a right femoral vein puncture and embolization of the hepatic artery were performed at a local hospital. Severe pain in the right lower extremity occurred 2 days after the operation.

Physical examination showed swelling and pain in the right lower extremity, pitting edema, elevated skin temperature, and palpable dorsal pedal pulse in the right lower extremity. However, no obvious abnormality in the left lower extremity, and other physical examinations were found.

**Figure 1.**
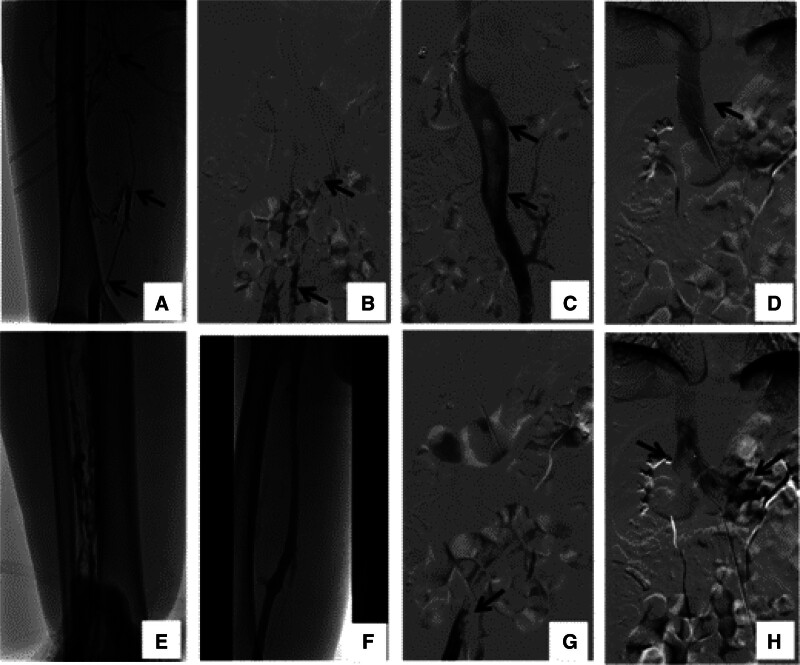
Contrast CT confirms the left-side IVC and no pulmonary artery embolism. (A) CTA showed iliac vein, iliac artery and left-sided IVC. (B) The IVC was left of the abdominal aorta. (C,F) Pulmonary CTA showed no pulmonary embolism. (D) The renal vein across the abdominal aorta. (E) The abdominal aorta on the left and the inferior vena cava on the right. CT = computed tomography, CTA = computed tomography angiography, IVC = left-sided inferior vena cava.

**Figure 2.**
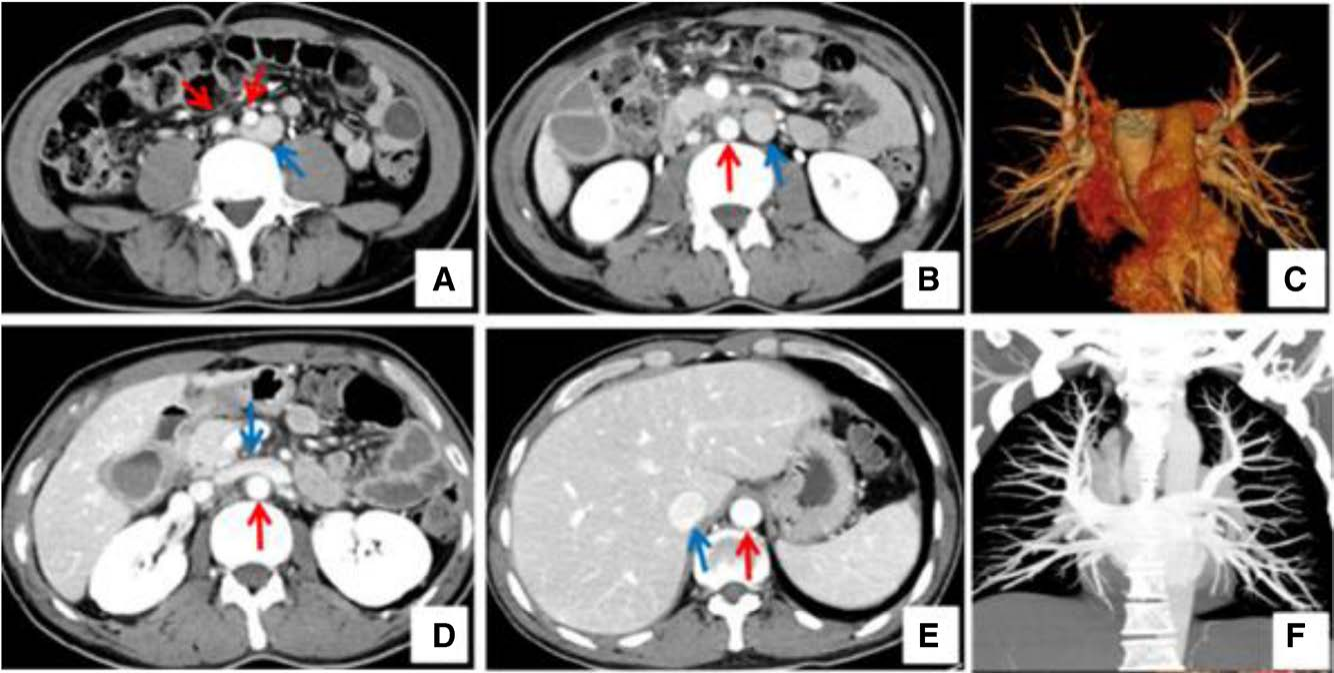
The right femoral-iliac vein thrombosis and inferior vena cava floating thrombosis were diagnosed by the DSA. (A–C) The right femoral-iliac vein thrombosis and inferior vena cava floating thrombosis were diagnosed by the DSA. (B and D) The IVC filter was placed above the renal veins under the guidance of venography. (E) Angiojet thrombus aspiration system was used to aspirate thrombus from the access of the anterior tibial vein. (F) Angiography showed that the thrombosis in distal iliac vein and femoral vein had disappeared. (G) Unifuse thrombolytic catheter was placed in IVC and proximal iliac vein. (H) Modified the position of IVCF to the IVC between the renal vein. IVC = Inferior vena cava, IVCF = inferior vena cava filter, IVC = left-sided inferior vena cava.

**Figure 3.**
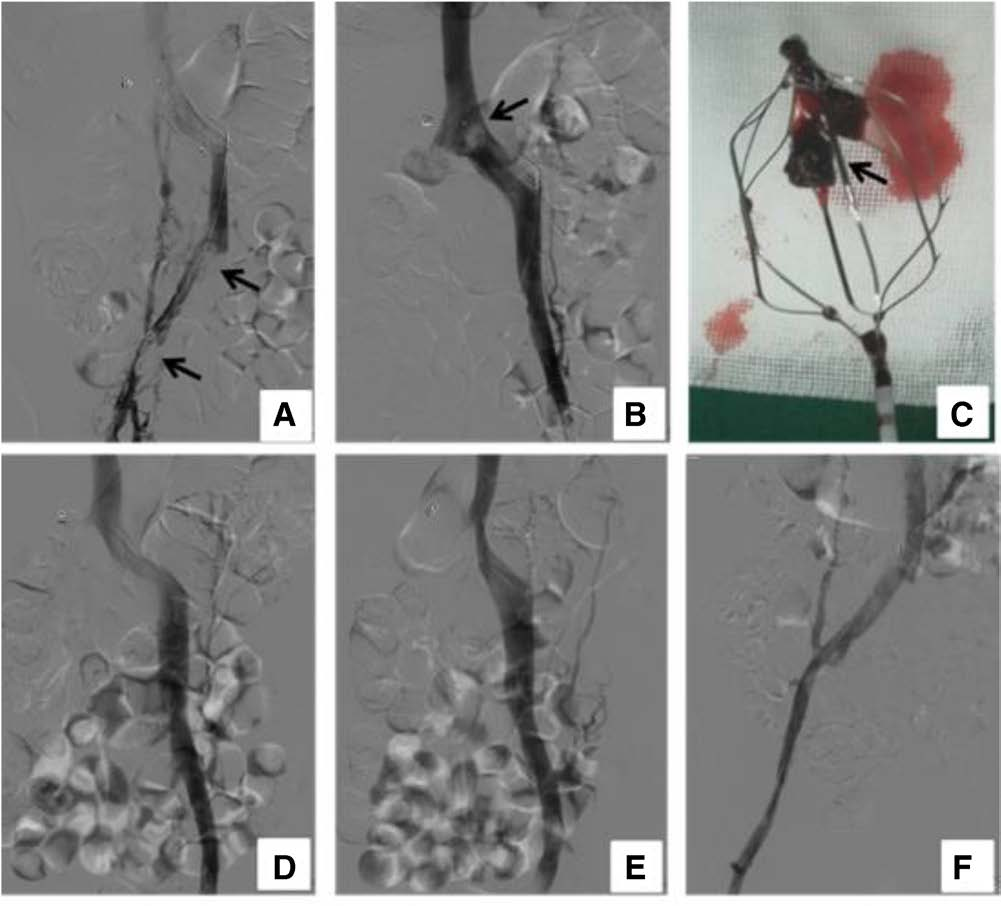
The thrombus was aspirated by an Angiojet thrombus aspiration system. Thrombosis in the iliac vein, femoral vein and inferior vena cava of the right lower extremity was basically eliminated. (A) There was still some thrombosis in the proximal iliac vein and inferior vena cava. (B) Angiography showed that IVC floating thrombus had disappeared, the proximal iliac vein still had some thrombus and part of the thrombus adhered to the filter. (C) IVC filter was removed. Some thrombus was trapped within the filter. (D–F) The right iliac vein and femoral vein thrombus were dissolved. IVC = left-sided inferior vena cava.

After admission to our hospital, a venous duplex ultrasound showed right venous thrombosis in the lower extremities. The biochemical examination revealed elevated D-dimer, and Contrast CT confirmed the left-sided IVC (Fig. 1A, B, D, E) and no pulmonary artery embolism (Fig. 1C, F). Therefore, we suspected acute right lower extremity venous thrombosis and a left-sided IVC. The venography after the puncture of the dorsal foot vein showed acute thrombosis of the right lower extremity deep vein. The IVC angiography showed a Left-sided IVC thrombosis combined with a large floating thrombus (Fig. 2A–C). To prevent thrombus detachment from IVC leads to pulmonary embolism (PE), the IVC filter was placed above the renal veins under the guidance of venography (Fig. 2B, D). The angiojet thrombus aspiration system was used to aspirate the thrombus from the access of the anterior tibial vein (Fig. 2E). After the catheter was passed through the anterior tibial vein to the external iliac vein, intravenously, urokinase was sprayed through the catheter. Then the thrombus was aspirated out with a catheter. After moving the thrombolytic catheter twice, the angiography showed that the thrombus in distal iliac vein and femoral vein had disappeared (Fig. 2F). During the thrombus in proximal iliac vein and a partial thrombus in IVCF (Fig. 2G), one unifuse thrombolytic catheter was placed in IVC and iliac vein (Fig. 2G). The position of IVCF to the IVC between the renal vein was also modified (Fig. 2H). The patient felt severe back pain after the operation. On the second day, venous angiography was performed, which showed that the IVC floating thrombus had disappeared, the proximal iliac vein still had some thrombus and part of the thrombus adhered to the filter (Fig. 3A, B). Then, the inferior vena cava filter was removed, and we can see thrombus being trapped within the filter (Fig. 3C), and the patient continued to receive catheter thrombolysis in the iliac vein. On the third day, the Unifuse thrombolytic catheter showed that all right iliac and femoral vein thrombus were dissolved (Fig. 3D–F). Thus, the thrombolytic catheter was removed, but anticoagulant treatment was continued. The patient recovered well after surgery, with no postoperative complications, including bleeding at the puncture site, tenderness, or swelling of the right lower extremity. The patient was discharged from the hospital and instructed to wear medical compression stockings for a long time, take rivaroxaban orally and have regular reexaminations.

## 3. Discussion

The normal adult right-sided IVC is formed between the 4th and 8th weeks of gestation. The normal IVC consists of 4 main segments: hepatic, suprarenal, renal, and infrarenal. The development of these segments is rather a complex process.^[1]^ The most common anomalies of IVC are retro-aortic left renal vein, double IVC, left-sided IVC, and interruption of the IVC with azygos continuation.^[2]^ In our case, an enhanced CT revealed a left-sided IVC connected with both iliac veins, conjoining the normal right-sided suprarenal IVC (Fig. 1).

Although rare, the clinical significance of a left-sided IVC has been recognized by many scholars. Moreover, identifying a left-sided IVC is crucial in terms of surgical and interventional procedures planning to prevent unexpected venous injuries and eventful bleeding. Shunya Shindo et al reported that large venous anomalies carry a higher risk of injury during aortic surgery. Of note, patients with IVC and left renal vein anomalies were at particularly high risk during the aortic surgery.^[3]^

For radiologists, awareness of left-sided IVC is essential since it can be misdiagnosed as lymphadenopathy on imaging, leading to unnecessary chemotherapy.^[4]^

Many studies reported that congenital anomalies of IVC might predispose to venous thromboembolism, especially in young patients. This might occur due to the changes in blood flow induced by the anomalous IVC or compression of the vessel or its major tributaries.^[5]^ However, no evidence has been found that left-sided IVC was an independent risk factor for venous thromboembolism. In the present case, the 42-year-old male suffered a massive acute deep vein thrombosis (DVT) and IVC thrombosis, which may be explained by multiple factors, including the history of trauma, hepatic rupture, venous catheter, immobilization, and IVC anomalies.

In rare cases, DVT in patients with congenital IVC anomalies may be complicated with fatal conditions such as IVC thrombosis and pulmonary thrombosis, causing a high mortality rate. As reported, IVC thrombosis only occurs in 4-15% of patients with a confirmed DVT.^[6]^ Around 60% to 80% of patients with IVC thrombosis were associated with congenital IVC anomalies.^[7]^ Rudolph et al reported a case of a 24-year-old woman who suffered an acute thrombosis in a left-sided IVC and recurrent PE.^[8]^ In our patient, a contrast CT ruled out PE (Fig. 1C, F). Therefore, recognizing IVC anomalies is vital for managing DVT patients.

Lower extremity DVT is 3 to 8 times more likely to involve the left leg.^[9]^ However, our patient presented with right-sided femoral-iliac vein thrombosis. Other studies have also reported right-sided occlusion in association with left-sided IVC.^[8–10]^ Hence, congenital anomalies of IVC should be suspected when a patient with no significant medical history presents with a right-sided lower extremity DVT.

Recent studies have found that the progress of endovascular therapies provides a wide range of new treatment options for DVT, and intravascular therapy significantly improves the vascular recanalization rate. Percutaneous aspiration thrombectomy effectively treats acute DVT in the lower extremity and restores venous patency.^[11]^ However, there are no guidelines recommending the use of percutaneous mechanic thrombectomy (PMT) for treating inferior vena cava thrombosis. For those with life or limb-threatening thrombosis or significant thrombus burden, systemic (for PE) or catheter-directed thrombolysis in conjunction with mechanical thrombectomy can be considered in the acute phase of treatment.^[12,13]^ In the current case, as for the proximal vessels, considering the huge thrombus in the left-sided IVC and common iliac vein, if only thrombus aspiration like PMT was performed on them, it was likely to lead to thrombus detachment and related serious complications, including death. Also, the existing evidence was indicative of reduced risk of subsequent PE for patients who received IVC filters,^[14]^ and preparing for PMT, catheter directed thrombolysis (CDT) or thrombectomy is the relative indication in Chinese guidance for the treatment of deep vein thrombosis. Therefore, we used an inferior vena cava filter, and catheter-directed thrombolysis was performed on the inferior vena cava and common iliac vein after the aspiration thrombectomy for the femoral and external iliac vein thrombosis. And it offers a faster means of recanalizing a thrombosed vein.

Therefore, for patients with lower extremity deep venous thrombosis complicated with IVC thrombosis, PMT combined with CDT treatment is a feasible way to restore vein patency, maintain valve function, and improve quality of life.

## 4. Conclusion

The relatively low incidence of left-sided IVC does not diminish the significance of its identification. However, we underlined the importance of a thorough preoperative evaluation of IVC anomalies in young patients with acute right or bilateral extremity DVT. Meanwhile, in this case, according to the treatment guidelines for lower extremity deep venous thrombosis, we found that PMT combined with CDT is a safe way to treat acute thrombosis.

## Author contributions

**Data curation:** Wei Chen, Xixi Min, Kanghui Dai, Henan Zheng.

**Formal analysis:** Kanghui Dai.

**Writing – original draft:** Zhinan Ju, Jiehua Qiu.
